# Challenges in transfusion and the role of Thalidomide in E‐β‐Thalassemia—A case report

**DOI:** 10.1002/ccr3.3141

**Published:** 2020-07-30

**Authors:** Rizwan Javed, Vivek Radhakrishnan, Sabita Basu, Mammen Chandy

**Affiliations:** ^1^ Department of Clinical Haematology and BMT TATA Medical Center Kolkata India; ^2^ Department of Transfusion Medicine TATA Medical Center Kolkata India

**Keywords:** alloantibodies, blood transfusions, E‐β‐thalassemia, thalidomide

## Abstract

Hemoglobin E‐β‐thalassemia is a common β‐thalassemia that has a variable presentation from mild to severe life‐threatening anemia. We describe such a case, who presented with severe anemia and multiple allo‐antibodies. Our case illustrates the role of thalidomide in transfusion‐sparing therapies in patients with transfusion‐dependent thalassemia and challenges in the blood bank.

## Graphical abstract

The role of thalidomide in transfusion‐sparing therapy in a transfusion‐dependent E‐β‐thalassemia patient. Thalidomide acts as a fetal hemoglobin inducer and alleviates alloimmunization by immunomodulation.

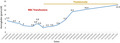

## 1 INTRODUCTION

Hemoglobin E‐β‐thalassemia (Hb E/β‐thalassemia) is a common β‐thalassemia with a clinical presentation varying from mild to severe life‐threatening anemia. Due to phenotypic variation, its management is tailored to maintain steady‐state hemoglobin by blood transfusions or drugs like hydroxycarbamide or both. Chronic blood transfusions are a risk to the patients, who may acquire transfusion‐transmitted infections, alloimmunizations, and iron‐overload.^1^ The use of fetal hemoglobin inducers like thalidomide in hemoglobinopathies reduces dependence on transfusions by inducing γ‐globin gene expression and erythroid cell proliferation.^2^

We report our experience with thalidomide therapy and the challenges in meeting the transfusion needs of a patient with E‐β‐thalassemia, who presented with severe anemia and multiple alloantibodies.

## 2 CASE REPORT

A 42 years old male patient, who was affected with thalassemia intermedia (E‐β‐thalassemia), presented to the outpatient department with complaints of constipation and weakness of 4 days duration. Since his diagnosis at the age of 16, he had received 9 RBC transfusions and had developed frontal bossing of skull. At 27 years, he was started on hydroxycarbamide and was stable in terms of hemoglobin levels (hemoglobin: 9 grams/dL, HbF: 62%). His present blood counts revealed severe anemia (hemoglobin: 5.3 grams/dL), and the treating physician requested for two units of packed red cell (RBC) transfusions. The patient received 15 RBC transfusions over 2 months with hemoglobin fluctuating between 5.3 and 2 gm/dL.

The bone marrow examination revealed erythroid hyperplasia consistent with hemoglobinopathy. The blood group of the patient was typed as “A” RhD(negative), and he was negative for Parvo B19 DNA PCR. On extensive immune‐hematological investigations by column agglutination technique (Ortho Biovue System, Ortho clinical diagnostics), anti‐Jka antibody was identified in the serum. The initial direct antiglobulin test and autocontrol were negative. Two units of cross‐matched, compatible, Jka antigen‐negative red cells were safely transfused. Since the patient required regular transfusions every week for 2 months, “A” RhD(negative) RBC was exhausted in the blood center and then “A” RhD(positive), Jka antigen‐negative red cells (11 units) were transfused. Subsequently, the patient developed post‐transfusion hemolysis (hemoglobin: 2 grams/dL, LDH: 1099 U/L) and anti‐D antibody was identified in the serum along with anti‐Jka. Thereafter, the patient was given either A or O Rh negative RBC (also negative for Jka antigen) transfusion.

**FIGURE 1:**
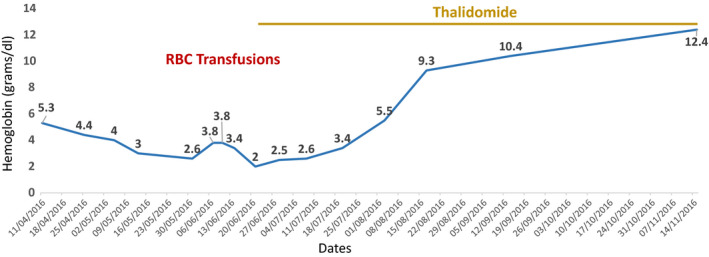
The patient was free of red cell transfusions within 2 mo of initiating oral thalidomide therapy, and the graph shows a rising hemoglobin value over 3 mo. The patient maintained a hemoglobin value above 12 gm/dL over 3 y till date

On initiation of thalidomide therapy (100 mg/day) along with weekly RBC transfusions (for 2 months), the patient's hemoglobin stabilized at 10.3 gm/dL in 3 months (see Figure 1). Bone marrow examination performed thereafter showed erythroid hyperplasia consistent with hemoglobinopathy. The patient's hemoglobin persistently improved and is consistently above 12 grams/dl over three years with HbF of 75%.

## 3 DISCUSSION

Thalassemia is a hereditary anemia resulting from defects in β globin gene. In 1976, it was first postulated that increased HbF benefits thalassemia patients by decreasing the imbalance between β and non‐β‐chains and consequently reduces hemolysis. Antoniani et al have used Clustered Regularly Interspaced short palindromic repeats (CRISPR) and CRISPR‐associated protein 9 (Cas9) (CRISPR/Cas9) system to disrupt γ‐δ intergenic HbF silencer, which leads to high γ‐globin in erythroblast and increased HbF synthesis.^3^ Many drugs have been studied as inducers of HbF for patients with β‐thalassemia and sickle cell disease. Hydroxycarbamide was found to be useful in moderate to severe forms of sickle cell disease and some cases of thalassemia intermedia.^4^ In our patient, hydroxycarbamide kept the patient stable for 15 years. As a histone deacetylase inhibitor, it improves the α/β chain imbalance typical of these disorders.^5^ The sudden drop in hemoglobin in our case is unclear. However, in the absence of Parvo virus infection (as Parvo B19 DNA PCR was negative), myelodysplastic syndrome and leukemia/lymphoma (ruled out by bone marrow examination and FISH), the severe anemia could only be explained by alloantibody delayed hemolytic transfusion reaction due to anti‐Jka antibody. The direct antiglobulin test was initially negative and, subsequently, became weakly positive after 9 Rh positive RBC transfusions (attributable to anti‐D antibody), leading to a drop in hemoglobin to 2 gm/dL and raised LDH.

Alloimmunization is a reaction of the immune system to foreign antigens and is one of the major complications of transfusions, particularly in patients who are chronically transfused. The most frequent antibodies (single or double antibodies) encountered in thalassemia are against Kell and subgroups of Rh. In a recent report, prevalence of double antibodies was 14% of the 50 cases studied, including Anti‐D + Anti‐C(8%), anti‐D + Anti‐E (2%), anti‐Kell + Anti‐D (2%), and anti‐Kell + KPa (2%). In our case, we identified anti‐Jka and anti‐D.^6^ A total of 29 units of RBC were transfused in the patient along with initiation of thalidomide therapy. Thalidomide may play an immunomodulatory role, in slowing erythroid maturation, increasing the proliferation of immature erythroid cells, and modulate hemoglobin transcription, resulting in induction of fetal Hb.^2^

Thalidomide is also postulated to reduce antibody production, besides suppressing NF‐KB induction by inflammatory cytokine. Tumor necrosis factor (TNF‐α), vascular endothelial growth factor (VEGF), and prostaglandin E2 synthesis (PG‐E2) are associated with increased release of reactive oxygen species (ROS) that stimulates P38 MAPK and results in increased HbF levels. Comparison of the effects of sodium butyrate and thalidomide in gene expression induction suggests higher capacity of the latter in increasing production of β‐ and γ‐globin genes.^5^, ^7^ Thalidomide and its derivatives (pomalidomide and lenalidomide) are beneficial in the treatment of sickle cell disease and other hemoglobinopathies. The monthly cost of generic thalidomide in India is around US$ 28. Since clinical experience is limited, it is not widely used and larger clinical trials are underway.^4^, ^8^, ^9^, ^10^ However, despite a high HbF induction capacity, it has limited application due to teratogenic effects and some neurological complications.^5^ Thalidomide is not prescribed to pregnant women. Thalidomide may also cause sensorimotor peripheral neuropathy, fatigue, and constipation. However, the adverse effects are dose and duration dependent, hence at a oral dose of 200 mg/day or less, it is well‐tolerated.^11^

Our case illustrates the role of thalidomide in transfusion‐sparing therapies in patients with transfusion‐dependent thalassemia and the challenges a transfusion medicine unit faces in providing safe transfusions. In conclusion, we suggest that further biological and clinical studies must be conducted to define the potential use of thalidomide in thalassemia. It may offer a cost effective treatment among the current available therapeutic modalities. In transfusion‐dependent patients, phenotype‐matched red cell transfusions are recommended, as this would minimize the problems of red cell alloimmunization.

## CONFLICT OF INTEREST

None declared.

## AUTHOR CONTRIBUTIONS

Rizwan Javed: conducted the literature search on the topic and drafted the initial version of the manuscript. Vivek Radhakrishnan: provided clinical inputs and critical revision of the manuscript for intellectual content. Sabita Basu: supervised transfusion laboratory work‐up and provided critical revision of the manuscript for intellectual content. Mammen Chandy: reported clinical information and reviewed the manuscript.

## CONSENT

Patient consent was taken.
